# Atrial fibrosis, atrial fibrillation susceptibility, and molecular remodeling in female goats with cardiac TGF-β1 overexpression

**DOI:** 10.1007/s11248-026-00517-5

**Published:** 2026-11-06

**Authors:** Misha Regouski, Jens Fillmore, Raquel Larsen, Cheyenne Marriott, Tayler Patrick, Alexis Sweat, Rusty Stott, Aaron Thomas, Arnaud Van Wettere, Michael J. Cutler, Heloisa M. Rutigliano, Irina A. Polejaeva

**Affiliations:** 1 Department of Animal, Dairy, and Veterinary Sciences, Utah State University, Logan, UT USA; 2 Department of Veterinary Clinical and Life Sciences, College of Veterinary Medicine, Utah State University, Logan, UT USA; 3 Intermountain Medical Center, , Intermountain Medical Center Heart Institute, Murray, UT USA

**Keywords:** Atrial fibrillation, Atrial fibrosis, TGFß-1, Large animal models

## Abstract

Atrial Fibrillation (AF) is the most common cardiac arrhythmia in adult humans. Atrial fibrosis plays a primary role in developing and sustaining this disease phenotype. Specifically, TGF-β1, a potent pro-fibrotic molecule, is implicated in cell turnover, increasing the amount of extracellular matrix in the heart. This increase leads to aberrant electrical signaling and can produce AF. In this study, we hypothesized that TGF-β1 modulates AF susceptibility in TGF-β1 transgenic goats and that genes related to TGF-β1 and AF would be differentially expressed when compared to wild-type (WT) controls. Transgenic (TG) female goats expressing the active form of human TGF-β1 under the control of the cardiac-specific α-myosin heavy chain (α-MHC) promoter and age-matched wild-type female goats were utilized in the study. Pacemakers were implanted in all animals for continuous arrhythmia monitoring and AF inducibility, which was evaluated using five separate 10 s bursts of atrial pacing (200–160 ms). Tissue samples were collected from both atria of TG and WT goats. RNA was extracted, and gene expression analyses were run on a selected panel of genes. Immunohistochemistry was performed on atrial tissue to evaluate for the presence of selected inflammatory markers to elucidate AF substrate in these animals. There was a significant increase in AF inducibility in both frequency and duration in TGF-β1 goats compared to their WT counterparts. There was no significant difference in the number of inflammatory cells tested by immunohistochemistry. Differentially expressed genes between the TG and WT groups were found in several functional categories including AF, apoptosis, extracellular matrix, inflammation, and TGF-β1.These data support the hypothesis that fibrosis is an important modulator of AF susceptibility. More specifically, an overexpression of TGF-β1 significantly increases expression levels of ECM proteins and TGF-β1 signaling modulators, adding to elevated AF risk without altering underlying inflammation.

## Introduction

The development of transgenic large animal models of atrial fibrillation (AF) provides a unique platform to study cardiac fibrosis and its implications on AF. To better understand the molecular underpinnings of AF, animal models can provide crucial data at the tissue and cellular levels without the complex ethical issues associated with human studies. Human cardiac tissue is often difficult to procure as typical availability includes only the atrial appendage, which is small and may not be indicative of whole chamber conductivity, or tissue that has been metabolically inactive for an extended period of time. Recent advancements in in vivo imaging have progressed in their usefulness but still have limitations in understanding molecular mechanisms of AF pathology (Olsen et al. 2016). This leaves space for utilizing new technologies to produce large animal models that can provide adequate tissue and imaging capabilities which can then be translated to human medicine.

Our group developed a transgenic (TG) goat model of cardiac fibrosis. Fibrosis is a substrate in the pathogenesis of AF as a fibrotic substrate fosters aberrant electrical conductivity, which is thought to contribute to the phenotype (Frangogiannis et al. 2019). AF occurs when the atria develop rapid disorganized electrical activity and this can develop secondary to structural changes such as fibrotic patches in the heart leading to aberrant electrical stimulation and ectopic activity of atrial myocytes causing spontaneous depolarization (Brundel et al. 2022). This spontaneous depolarization causes portions of the atria to randomly contract out of concert with itself and the ventricles. Significant research has focused on aberrant electrical activity caused by structural remodeling, such as fibrosis. As such, when developing this TG goat model, progressive fibrosis was paramount to mimic the human progression of the disease (Polejaeva et al. 2016). Although TG goats exhibit higher AF susceptibility compared to wild-type (WT) controls, further information at the molecular level is needed to understand the role fibrosis plays in AF.

Previously, this model has been shown to exhibit spontaneous AF when exposed to environmental stressors, such as endurance exercise (Regouski et al. 2019). Additionally, ex-vivo cardiac magnetic resonance imaging studies have shown slower atrial conduction speeds and disorganized fibrotic patches when compared to WT control goat hearts (Kwan et al. 2024). Thus, several studies evaluating the role of fibrosis in this goat model have shown an increased AF susceptibility, and even episodes of spontaneous AF, but expression of key genes associated with fibrosis has not been evaluated. Therefore, understanding key molecular pathways involving inflammation and TGF-β is crucial to gain further understanding into this model’s capabilities of recapitulating AF with underlying fibrosis as the substrate.

The crux of using TGF-β1 as a modulator of cardiac fibrosis lies in its ability to promote fibrosis while also being anti-inflammatory. Routine insult to tissue typically results in higher local and circulating inflammatory markers (Wynn et al. 2012). However, TGF-β1 overexpression results in higher fibrosis by inducing differentiation of fibroblasts into myofibroblasts and secreting excessive amounts of ECM (Bolivar et al. 2021) while maintaining an overall global anti-inflammatory effect. Investigating the link between non-inflammatory fibrosis in the atria and AF occurrence can determine if fibrosis alone is enough to sustain AF. To generate this model, we produced genetically modified goats via somatic cell nuclear transfer (SCNT) using cells containing a cardiac specific overexpression of TGF-β1. This modification leads to diffuse fibrosis in the atria (Polejaeva et al. 2016).

The present study aimed to compare the expression of genes related to the TGF-β1 pathway, inflammation, fibrosis, apoptosis, and atrial fibrillation, the presence of inflammatory cells and the occurrence of AF between TG goats and their WT counterparts to help elucidate the role cardiac fibrosis has on the molecular and electrophysiologic properties of the TG goat heart. We hypothesized TG goats would have an upregulation of transcript levels in most gene categories, with the exception of genes related to inflammation as TGF-β1 has anti-inflammatory properties (de Oliveira et al. 2021). This molecular and cellular profiling may help in understanding the role that fibrosis plays in the pathogenesis of AF in this model and may glean insight into clinical cases of AF with a fibrotic cause.

## Materials and methods

### Animals

Procedures were approved and conducted by the published guidelines by Utah State University Institutional Animal Care and Use Committee (IACUC). Twelve female adult domestic goats (*Capra hircus)* between two and seven years of age were used in this study. Female goats were selected as they comprised the animals available for the use of this study, the animals had previously been bred and had successfully kidded, though were more than one year post-parity at the time of experimentation. Data collection was performed during anestrous. The six TG animals used have overexpression of human TGF-β1 in atrial tissue and come from a Nubian Boer cross background. The six WT goats used were Spanish Nubian crosses. The generation of human TGF-β1 transgenic goats is described in detail by Polejaeva et al. (Polejaeva et al. 2016). Briefly, a cardiac specific α-myosin heavy chain (αMHC) promoter was subcloned into the pcDNA3.1D vector. The Invitrogen NEON transfection (Invitrogen, Waltham, MA, USA) was used to electroporate primary goat fetal fibroblasts. Cells containing the transgene were used for SCNT and were screened by polymerase chain reaction (PCR) to confirm the presence of the transgene. Oocytes were collected as described elsewhere (Yang 2016). SCNT, recipient synchronization, and embryo transfers were performed as described by our laboratory team (Yang et al. 2018). Once recipients gave birth, clones were screened via PCR to ensure the construct was wholly intact and allowed to mature to at least 18 months of age before being utilized on any study. Our group has characterized this model previously, showing an increased P wave duration, increased ECM in the atria, and inducible AF episodes (Polejaeva et al. 2016; Regouski et al. 2019).

### Pacemaker implantation

Dual chamber pacemakers were implanted into the right atrium of TG and WT goats that were between two and seven years old. Anesthesia was induced in goats undergoing implantation with a combination of Ketamine (6 mg/kg) and Diazepam (0.1–0.2 mg/kg) administered intravenously (IV) via the jugular vein. Immediately following induction, animals were intubated, and anesthesia was maintained with isoflurane dosed to effect (2–4%) in inspired oxygen. The surgical site was scrubbed with three alternating washes of a chlorhexidine scrub followed by isopropyl alcohol. As part of the preparation, each animal received Cefazolin, 20 mg/kg, IV intraoperatively to reduce the risk of bacterial infection arising during the procedure, as well as a lavage of cefazolin at the generator implant site. Pacemakers were generously donated by Boston Scientific, and either their Accolade or Altrua model platform (Boston Scientific, 300 Boston Scientific Way, Marlborough, MA) was used. All animals received the same lead configuration and identical programmed burst pacing program parameters. AF induction was therefore performed using the same experimental pacing protocol across animals. Pacemaker leads were introduced into the right atria through a cutdown to the right jugular vein. The leads were tunneled subcutaneously and attached to a pacemaker generator in a separate site on the neck. Leads were visualized using C-arm fluoroscopy for correct orientation and insertion into the right atrial wall. Conductivity was tested immediately post-implantation and any lead revisions were performed as necessary. The incision was closed, and the animals were carefully monitored during recovery. Animals were provided with post-operative analgesia by a single injection of flunixin meglumine (1.1 mg/kg administered IV). Standard postoperative care such as bandage checks and antibiotic administration was performed daily until the incisions healed. Lead stability was tested one-week post-procedure, and revisions were performed as necessary. Although no goats experienced post-operative infections and incisions were healed within a week, no study was started until two months post-surgery.

### AF induction

Burst pacing for 10 s at varying milliseconds (160–200) was performed: once at 200 mS, two at 180 mS, and two at 160 mS. AF inducibility was assessed by recording the number of AF episodes and their durations at each burst. Animals were monitored throughout the pacing procedure and were permitted to self terminate induced AF episodes. No spontaneous episodes were observed during the baseline experimental phases. AF induction was performed as part of the approved experimental protocol. TG goats assessed for AF inducibility were all between four and seven years of age and had age-matched WT animals as a comparison. All animals weighed between 45 and 73 kg. Baseline inducibility was performed twice two weeks apart on each goat.

### Sample collection

Goats were euthanized according to current American Veterinary Medical Association guidelines with an intravenous injection of phenobarbital at a 20 mg/kg dose. Hearts were excised from the chest immediately upon confirming the lack of heartbeat and corneal response. Cardiac muscle tissue from left and right atria (LA and RA) were snap-frozen in liquid nitrogen for RNA isolation. Additionally, the same tissue types were placed into optimal cutting temperature compound (OCT) in plastic boats submerged in chilled isopentane and stored at − 80 °C as well as formaldehyde for histological analysis. Reagents detailed below were all obtained from Thermo Fisher Scientific (*Thermo Fisher, Waltham, MA)* unless otherwise noted.

### Immunohistochemistry

Atrial samples collected in OCT for immunohistochemistry (IHC) staining were processed using an in-house protocol (Koroghli et al. 2018). Briefly, sections were removed from storage and allowed to air dry, sections of right atrium from each animal were cut at 20 μm on a cryostat, placed on glass slides and then stained for different immune cell populations. Antibodies were obtained from Washington State University’s Monoclonal Antibody Center (WSU, Pullman WA) unless otherwise noted. Antibodies included MUC2A used at a 1:200 dilution for the CD2 antigen, 17D used at a 1:200 dilution for CD4 antigen, GB25A used at a 1:400 dilution for CD21, and finally, TH14B used at a 1:200 dilution for MHC-II antigen. Ten slides per cardiac chamber per animal were saturated with a 0.3% hydrogen peroxide solution, washed in phosphate-buffered saline (PBS), and then blocked in a 2% normal goat serum and 1% bovine serum albumin solution. Then, each slide set was incubated with a primary antibody at the working concentrations described above and rinsed in PBS. Incubation with a secondary antibody (biotinylated goat anti-mouse IgG [Vector Labs, Newark, CA, USA]) diluted in casein to 1:200 was introduced to the slides, after incubation, slides were rinsed in PBS. Streptavidin conjugate was placed on the slides, rinsed in PBS, and AEC chromogen was applied. Slides were rinsed in distilled water and then counterstained with Gill’s #2 hematoxylin. Fluoromount G was applied, coverslips were placed and sealed with clear nail polish. Slides were then imaged using a Zeiss Axio Observer Z1 (Zeiss, Hebron, KY, USA), ten fields of view at 40 × objective were visualized for the presence of positive cells from antibody staining and the number of positive cells per area was recorded.

### Histology

Formalin-fixed heart sections were collected, processed, and embedded in paraffin following routine histological techniques. 5 μm thick sections were stained with Masson’s Trichrome stain according to standard methods and evaluated under light microscopy. Images were obtained using an Olympus BX 43 microscope with a 40X objective. Fibrosis amounts were determined by measuring the area occupied by blue staining (collagen) using Photoshop. A manual threshold was set and those settings were used on all images to determine the area percent of fibrosis.

### Gene expression

The Fluidigm Biomark system (Standard Biotools, San Francisco, CA, USA) was used for gene expression analysis. Snap frozen tissue was used from each atrium for RNA extraction. Purified RNA samples were converted to cDNA for use in this manner as follows. The RNeasy mini kit (Qiagen, Germantown, Maryland, USA) was used according to the manufacturer’s protocol. A QIAzol/chloroform purification step was added as the nature of cardiac tissue proved difficult for high-quality RNA isolation without this additional step. Tissue pieces weighing 20 mg ± 2 mg from both atria from all animals were placed into QIAzol (Qiagen, Germantown, Maryland, USA) and homogenized. Chloroform was added, and the samples were spun to separate out total RNA. The samples were then placed into spin columns provided in the kit, ethanol was added, and several wash steps followed. Finally, RNA was eluted in RNase-free water. Sample concentration was determined using a Nanodrop 2000 spectrophotometer.

RNA samples were diluted to 0.5 μg/μL in DNA suspension buffer and converted to cDNA using a VILO cDNA synthesis kit wherein diluted mRNA was mixed with the proprietary superscript master mix enzyme and incubated on a thermocycler for 10 min at 25 °C, 10 min at 50 °C, and five minutes at 85 °C. Primers were designed using NCBI blast data and efficiency was determined using stepwise dilutions from 1:100 up to 1:10000. Primers and samples were run in duplicate, producing four replicates of each sample/primer combination. Primer sequences and efficiencies can be found in Table 1, efficiencies lower than 85% were excluded.

**Table 1:** Genes selected for real-time qPCR analysis, their functional categories, primer sequences and efficiencies

Gene	Gene Name	Primer Sequence	Efficiency
TGFβ Signaling Genes			
*ALK1*	Activin receptor like kinase 1	TGCTGCTCAGACACGACAA CTCGAGTTGCGGGAAGTCA	95%
*ALK3*	Activin receptor like kinase 3	TGGCAGTGGATCTGGACTAC CTTTACCAACTTGCCGAACCA	94%
*BMP4*	Bone morphogenic protein 4	GGCATCGGTCTGGAGTATCC GGTCCCTGGGATGTTCTCC	94%
*BMP6*	Bone morphogenic protein 6	CGACATCCCTTCTCCTCAAGAA AGGGGAGAACTCCTTGTCGTA	95%
*hTGFβ*	Human transforming growth factor beta	GCGTGCTAATGGTGGAAACCCA GCTGTGTGTACTCTGCTTGAAC	86%
*LOX*	Lysyl Oxidase	TGGTGCCCGATCCCTACTAC GCAGCGCATCTCAGGTTGTA	91%
*LTBP2*	Latent transforming growth factor beta binding protein 2	GACGGGTGACTGCATTGACA CCGTCGGTGTTGTCACAGAA	95%
*LTBP3*	Latent transforming growth factor beta binding protein 3	GATGGCACTGCTGCCTTCA GTGGGAGGTGAGGATGTGGTA	95%
*MKP1*	Map kinase phosphatase 1	TTGCTGATGTGCCAGACCAA GCCGTTGACACACCATTTCC	93%
*SMAD2*	Mothers against decapentaplegic homolog 2	ACCACTACCAGAGAGTTGAGAC GGGCAGTTCTGTTAGGATCTCA	94%
*SMAD3*	Mothers against decapentaplegic homolog 3	AGCCCCAGAGCAATATTCCA GTGGTTCATCTGGTGGTCAC	94%
Extra Cellular Matrix Genes			
*COL1A1*	Collagen type I alpha 1 chain	TGGTGCTGACGGACCTGCT CACACCACGCTGTCCAGCAA	80%
*COL1A2*	Collagen type I alpha 2 chain	CTGGTGAGCCTGGTGAACCT CATCCTCACCAGCCTTGCCA	96%
*COL3A1*	Collagen type III alpha 1 chain	CACGCAAGGCCGTCAGACTA AGGGCCAATGTCCGCACCA	95%
*FN1*	Fibronectin 1	CACACCCTTGACTCCTGTTGT CTCTTCACTCAGCTCATACTCCA	89%
*MMP2*	Matrix metalloprotein 2	CCATGATGGAGAGGCTGACA GCCCGTCTTTGCCATCAAA	89%
Apoptosis			
*BAX*	BCL 2 like protein	TGGCGGCTGAAATGTTTTCC CCAGTTTGCTGGCAAAGTAGAA	89%
Inflammation Genes			
*CD105*	Endoglin	CCACCCCAATGAACCTTCAAAC TACAGGGCAGTGGACTCTCA	95%
*IFNg*	Interferon gamma	GATAACCAGGTCATTCAAAGGAGC GATCATCCACCGGAATTTGAATC	93%
*IGF2*	Insulin like growth factor 2	CCGCTGTTCGGTTTGCAATA TGATCCCCATTGATGTCTGGAA	94%
*IL1*	Interleukin 1	GCCTTCAATAACTGTGGAACCAAT GTATATTTCAGGCTTGGTGAAAGGA	91%
*IL4*	Interleukin 4	GGCGTATCTACAGGAGCCACAC CAAGAGGTCTTTCAGCGTACTTGT	92%
*IL5*	Interleukin 5	TGGTGGCAGAGACCTTGACA GAATCATCAAGTTCCCATCACCTA	93%
*IL6*	Interleukin 6	GGCTCCCATGATTGTGGTAGTT GCCCAGTGGACAGGTTTCTG	93%
*IL8*	Interleukin 8	GGAAAAGTGGGTGCAGAAGGT GGTGGTTTTTTCTTTTTCATGGA	86%
*IL10*	Interleukin 10	GAGCAAGGCGGTGGAGAAGG GATGAAGATGTCAAACTCACTCATGG	89%
*IL15*	Interleukin 15	GGGCTGTATCAGTGCAAGTCTTC ATTGGGATGAGCATCACTTTCAG	85%
*TNFα*	Tumor Necrosis Factor alpha	TCTACCAGGGAGGAGTCTTCCA GTCCGGCAGGTTGATCTCA	92%
Atrial Fibrillation Genes			
*CX43*	Connexin 43	GGGCTTGCTGAGAACCTACA TGTACCACTGGATCAGCAAGAA	94%
*KCNA1*	Potassium voltage-gated channel subfamily A member 1	AGCTGAAGGACGAGAAGGAC CGTGAAGATGTTGGCGTTGTA	94%
*KCNIP1*	Potassium voltage-gated channel interacting protein 1	CCTTCTCCTCTCTGCAAACC GCAAACCATGGTCATCTCCA	85%
*SHOX2*	Short stature homeobox 2	GGACCAATTTCACCCTGGAA GGCTCAGTTCTTCACGCATA	94%
*SNAIL1*	Zinc finger protein SNAI 1	TGCCCTCAAGATGCACATCC CCTTCCCGCAGGTGCTAC	91%
*TIMP1*	Metallopeptidase inhibitor 1	ACCGCAGAAGTCAATGAGAC AGGCACTGAACCCTTTGAAC	95%

### Statistical analyses

Statistical differences were assessed with a Student’s t-test for baseline inducibility between WT and TG animals after data was checked for normal distribution. For gene expression analyses, left and right atrial samples were evaluated as repeated measurements (using a repeated measures ANOVA test (Park et al. 2009)) within each animal, with genotype (TG vs. WT) representing the treatment effect and atrial chamber representing the repeated factor. The statistical comparisons reported in Figs. 5, 6, 7 and 8 represent the TG versus WT treatment effect. GAPDH and ACTB were used as reference genes based on their stable expression and amplification efficiency. For each sample, the mean expression of GAPDH and ACTB was used for normalization of the target transcript. Relative expression was subsequently normalized to the corresponding WT transcript level. Gene expression data are presented as log 2 fold-change relative to the reference gene and WT expression. A false discovery rate (FDR) test was applied to the gene expression data to correct for multiple comparisons (Benjamini and Hochberg 2018). Adjusted *p* values can be found in Table 2. Immunohistochemistry data was assessed using a Student’s t-test. A Mann–Whitney U test was used for histological analysis. All data are expressed as mean ± standard error of the mean (SEM). Statistical data analysis was performed using Jamovi (The Jamovi Project 2022). *jamovi* (Version 2.3).

**Table 2:** False discovery rate Analysis for genes analyzed via real-time qPCR

Gene	RM ANOVA *p* value	FDR adjusted *p* value	Rank
*KCNIP1*	0.0000000000010871	0.0000000000380485	1
*IL8*	0.0000000000062028	0.0000000001085490	2
*IL5*	0.0000000000068497	0.0000000000799132	3
*IL10*	0.0000000000071367	0.0000000000624461	4
*SHOX2*	0.0000000000091402	0.0000000000639814	5
*IFNG*	0.0000000000243010	0.0000000001417558	6
*SNAIL1*	0.0000000000264583	0.0000000001322915	7
*IL15*	0.0000000001478110	0.0000000006466731	8
*KCNA1*	0.0000000004016549	0.0000000015619913	9
*IL6*	0.0000000010741329	0.0000000037594652	10
*LTBP2*	0.0000000049462419	0.0000000157380424	11
*IL1*	0.0000000083553700	0.0000000243698292	12
*BMP6*	0.0000000777210000	0.0000002092488462	13
*TNFa*	0.0000001067615926	0.0000002669039815	14
*BMP4*	0.0000001126556000	0.0000002628630667	15
*SMAD3*	0.0000001593773135	0.0000003486378733	16
*TGFB*	0.0000002203534906	0.0000004536689512	17
*ALK3*	0.0000003158398000	0.0000006141329444	18
*TIMP1*	0.0000011403295448	0.0000021006070562	19
*SMAD*	0.0000014469365703	0.0000025321389980	20
*HTGFB*	0.0000026470000000	0.0000044116666667	21
*MKP1*	0.0000036115831614	0.0000057457004840	22
*ALK1*	0.0000038702000000	0.0000058894347826	23
*LTBP3*	0.0000038822575777	0.0000056616256341	24
*CD105*	0.0000062939700000	0.0000088115580000	25
*FN1*	0.0000071010000000	0.0000095590384615	26
*IGF2*	0.0000085583390000	0.0000110941431481	27
*MMP2*	0.0000088011883954	0.0000110014854943	28
*CX43*	0.0000166436000000	0.0000200871034483	29
*LOX*	0.0000200280145894	0.0000233660170210	30
*GTGFB*	0.0000545720000000	0.0000616135483871	31
*COL1A2*	0.0001259043000000	0.0001377078281250	32
*COL3A1*	0.0002685372970000	0.0002848122846970	33
*COL1A1*	0.0025351655000000	0.0026097291911765	34
*IL4*	0.7544071588200000	0.7544071588200000	35

A false discovery rate test was applied to genes used for qRT-PCR analysis. Initial *p* values are reported in addition to adjusted *p* values

## Results

### Inducibility

**Fig. 1:**
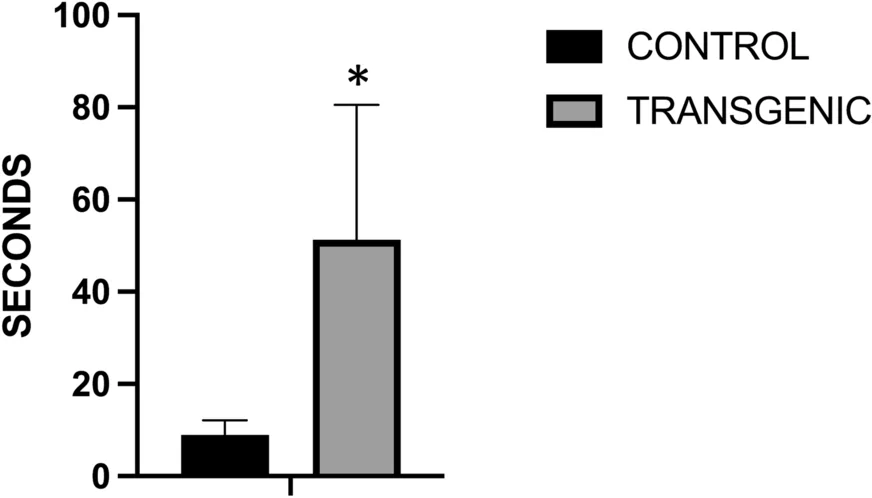
Average atrial fibrillation (AF) duration of TGF-β1 transgenic animals (TG, n = 6) when compared to their wild-type (WT, n = 6) controls. Average duration in seconds at baseline is significantly upregulated in TG animals when compared to their WT controls, as denoted by the symbol *. WT animals had an average induced AF duration of 8.98 ± 3.18 s, whereas TG animals had an average of 51.30 ± 29.27 s (*p *= 0.04*)* when analyzed using a Student’s t-test*.* Data are expressed as mean ± SEM

Because atrial fibrosis is expected to provide a structural substrate for AF, we first evaluated whether TGF-β1 overexpressing goats exhibited increased susceptibility to induced AF. A significant difference using a Student’s t-test between the inducibility of TG and WT animals was found with a *p*-value of 0.04 (Fig. 1). TG goats, on average, had episodes lasting 51.30 ± 29.27 s, whereas WT goats only had episodes lasting 8.98 ± 3.18. This shows that the TG animals have a higher level of AF susceptibility than their age and sex-matched WT controls.

### Immunohistochemistry and Masson’s Trichrome staining

**Fig. 2:**
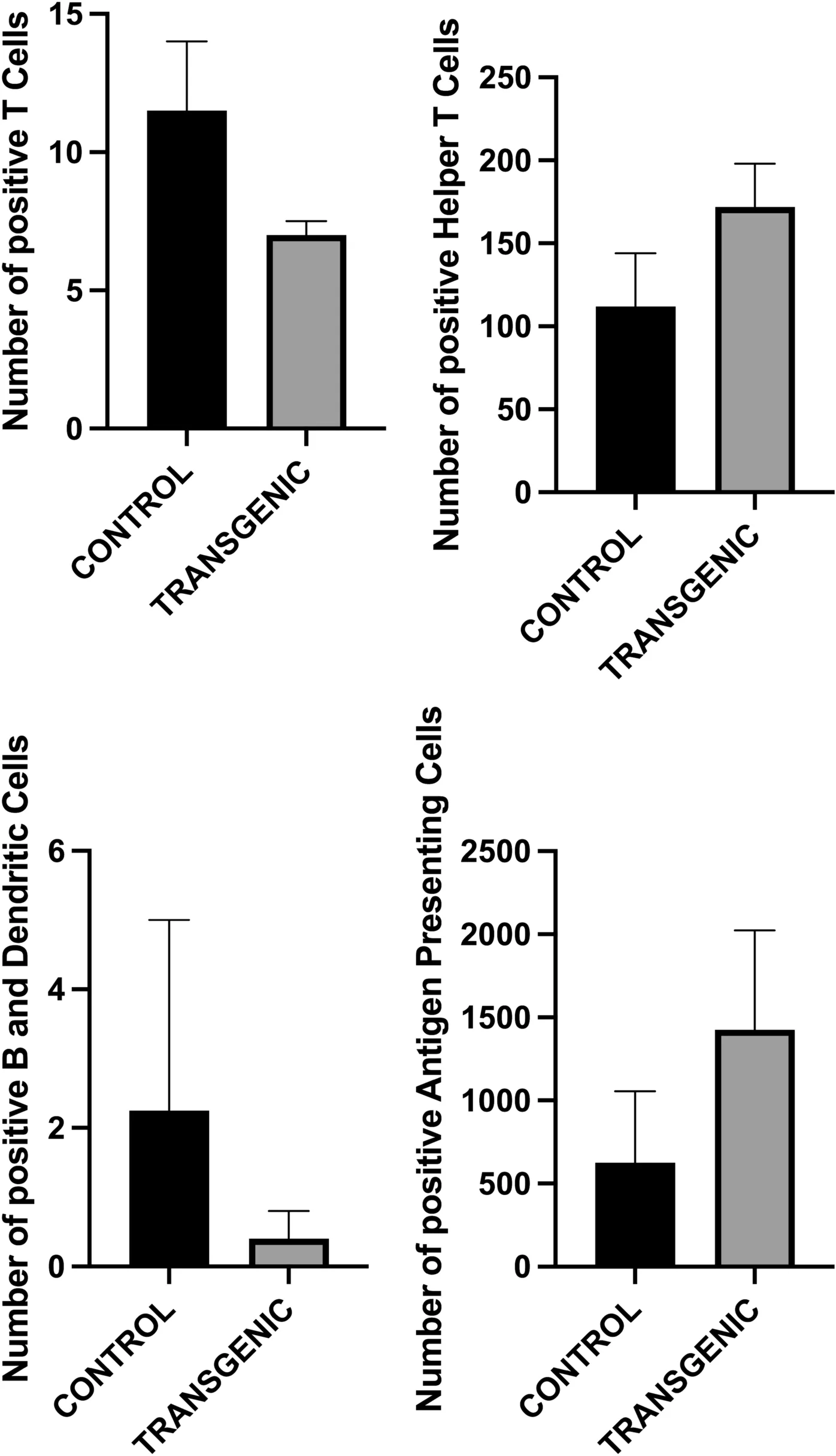
Analysis of immune cell populations in the right atrium of human TGF-β1 transgenic (TG, n = 6) goats compared to age and sex matched wild-type (WT, n = 6) control goats via immunohistochemistry staining. No significant differences were found between groups using a Student’s t-test. The number of T cells (CD2 + ) had a significance level of *p *= *0.19,* the number of T helper cells (CD4 + ) had a significance level of *p *= *0.13,* the number of B cells (CD21 + ) had a significance level of *p *= *0.36,* and finally, the number of antigen-presenting cells (MHC-II + ) had a significance level of *p *= *0.39.* Data are presented as average ± SEM

**Fig. 3:**
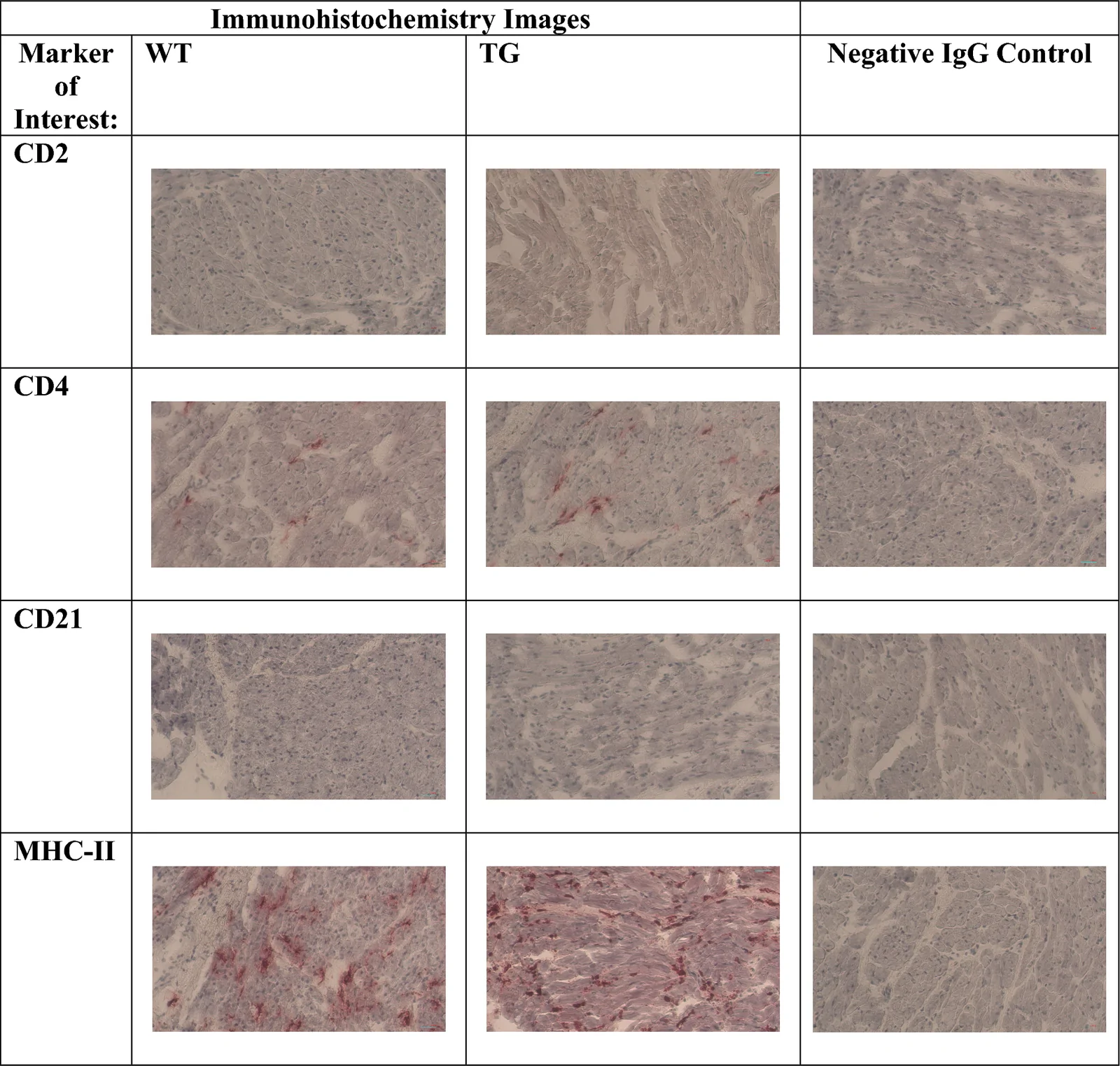
Immunohistochemical labeling of cells for CD2, CD4, CD21, and MHC-II in the atrium of TGF-β1transgenic (TG) and age and sex matched wild-type (WT) control goat atrial samples. Negative isotype-matched non-specific controls (Negative IgG control) were used. Using a Zeiss Axio Observer Z1 (Zeiss, Hebron, KY, USA) microscope, ten fields of view at 40 × objective were visualized for the presence of positive cells from antibody staining and the number of positive cells per area was recorded. Representative negative IgG control sections are shown to demonstrate nonspecific staining

**Fig. 4:**
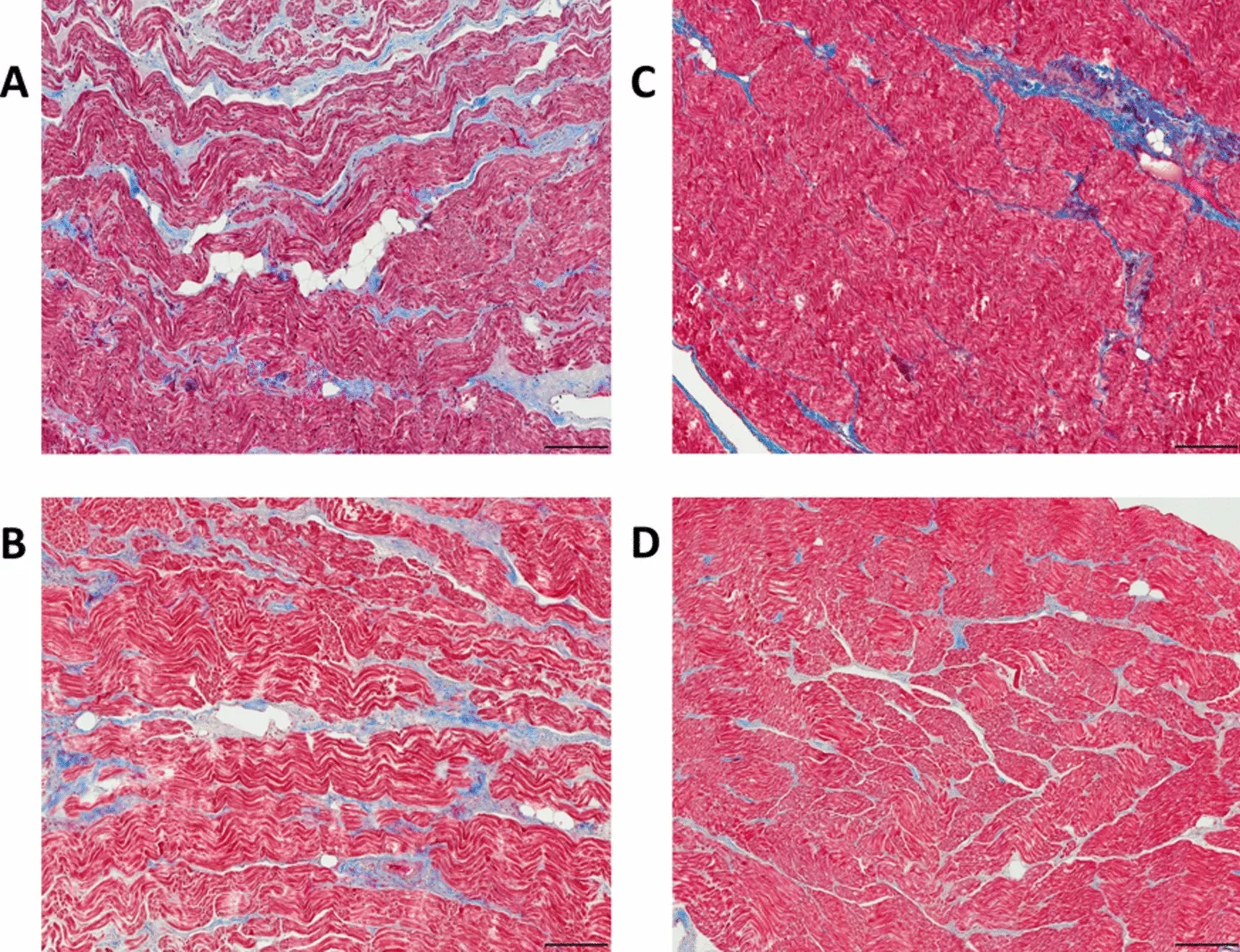
Representative images of Masson’s Trichrome staining of atrial tissue from two TGF-β1transgenic goats in panel A and B and two age and sex matched wild-type control goat atrial tissue in panel C and D. 40 × scale bar = 500 μm. Images were analyzed using Photoshop to obtain the percent fibrosis. Using the Mann–Whitney U test, percent fibrosis was significantly higher in TG hearts (TG, n = 8) compared to WT controls (WT, n = 7): median [IQR]: TG 6.5% (4–9%), WT 2% (0–2%), U = 56, *p *= 0.0014

Since TGF-β1 can promote fibrosis while exerting immunomodulatory effects, we evaluated whether the increased atrial fibrosis observed in TG goats was accompanied by increased immune cell infiltration. As shown in Figs. 2 and 3, there were no significant differences between TG and WT goats in the number of inflammatory cells (CD2 [T cells], CD4 [helper T cells], CD21 [B cells], and MHC-II [antigen-presenting cells] in the atria. Figure 4 shows representative images of Masson’s Trichrome staining of TG and WT atria where a significant difference in percent fibrosis between groups was observed.

### Gene expression

To further characterize the molecular pathways associated with the fibrotic and electrophysiologic phenotype, we evaluated mRNA transcripts involved in TGF-β1 signaling, ECM remodeling, inflammation, apoptosis, and AF. Several genes were significantly differentially expressed in the TG group when compared to the WT group of goats using repeated measures ANOVA. Many differences were found in varying functional categories, including genes implicated in AF, TGF-β1 pathway, ECM, and inflammation. No differences were found in the apoptotic gene category. Glyceraldehyde−3-phosphate dehydrogenase (GAPDH) and actin beta (ACTB) were used as housekeeping genes to normalize genes of interest to. These two genes were selected for their stable expression and efficiency determined by cycle thresholds between 23 and 28 and efficiencies of 94% and 92% respectively.

**Fig. 5:**
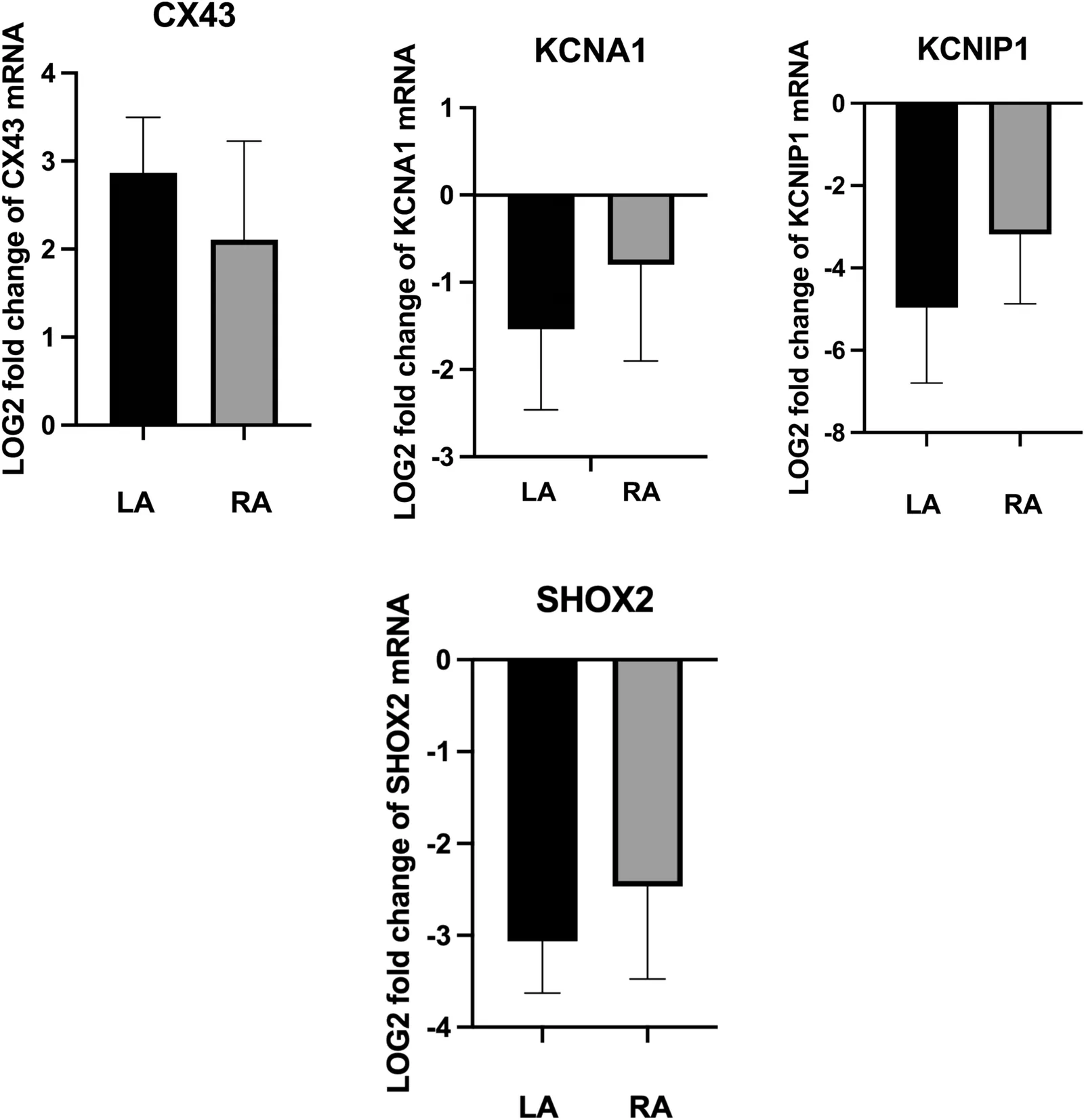
Log2 relative expression of atrial fibrillation (AF) related genes in atrial samples showing significant difference between TGF-β1 transgenic (TG; n = 6) and age and sex matched wild-type (WT; n = 6) control goat left (LA) and right (RA) atrial samples. Graphs represent 2^-ΔΔCT fold change in relative mRNA transcript levels in TG animals detected using qRT-PCR, target transcript expression was normalized to the mean mRNA transcript level expression of glyceraldehyde−3-phosphate dehydrogenase (GAPDH) and actin beta (ACTB) and subsequently to the corresponding WT transcript level for the individual target gene. After a false discovery rate adjustment of significant *p* values, and a fold change cut off of 1.5 applied: CX43 has a significance level of *p *= *0.003 KCNA1 p *= *0.012, KCNIP1 p *= *0.009, SHOX2 p *= *0.02.* Non-adjusted *p* values can be found in Table 2. *P* values reflect differences between TG and WT animals within a given gene and heart chamber. Data are expressed as mean ± SEM. Statistical significance represents the genotype/treatment effect comparing TG and WT animals; atrial chamber was included as a repeated factor in the analysis

**Fig. 6:**
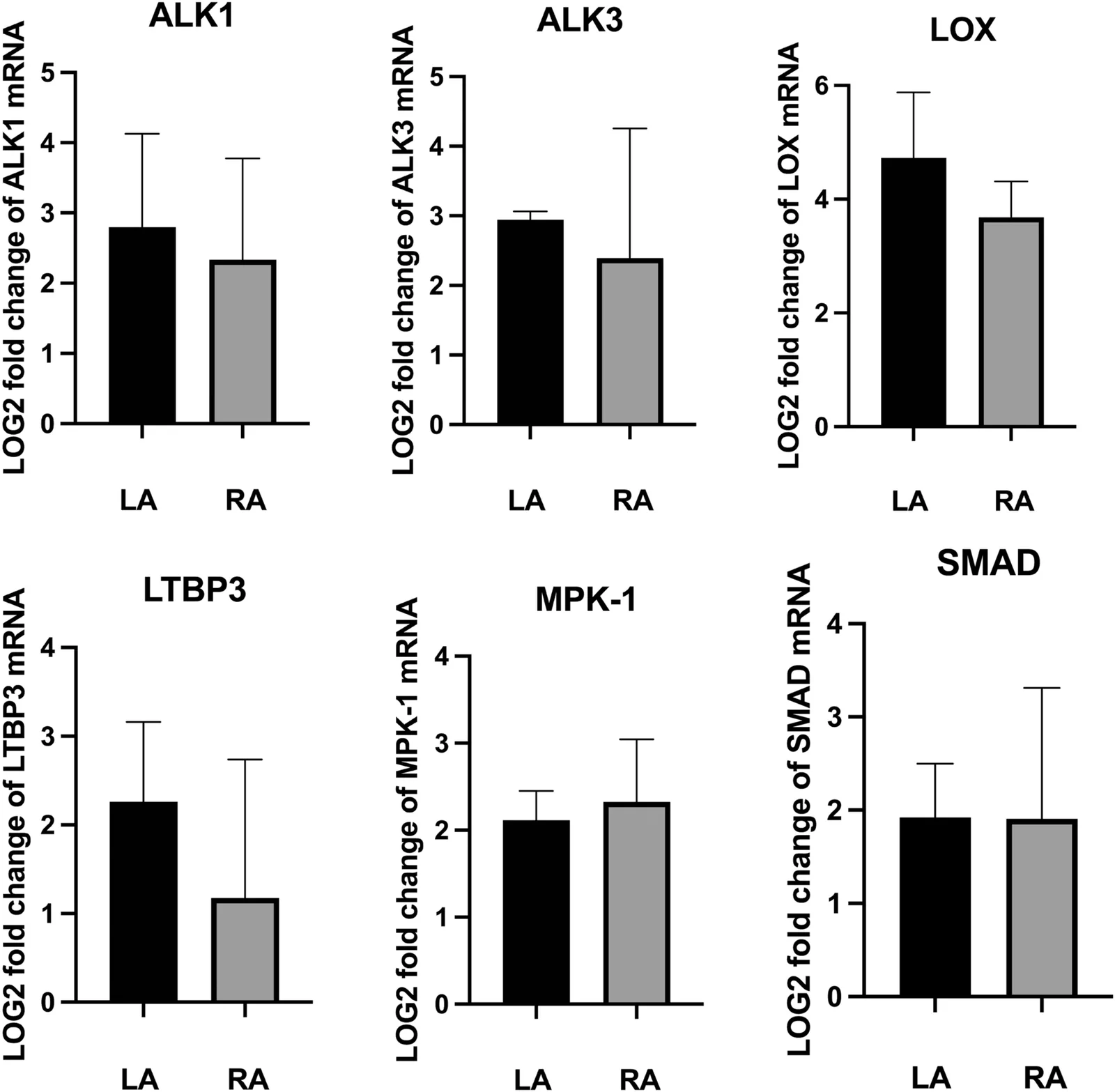
Log2 relative expression of TGFβ-1 related genes in atrial samples showing significant difference between TGF-β1transgenic (TG; n = 6) and age and sex matched wild-type (WT; n = 6) control goat left (LA) and right (RA) atrial samples. Graphs represent 2^-ΔΔCT fold change in relative mRNA transcript levels in TG animals detected using qRT-PCR, target transcript expression was normalized to the mean mRNA transcript level expression of glyceraldehyde−3-phosphate dehydrogenase (GAPDH) and actin beta (ACTB) and subsequently to the corresponding WT transcript level for the individual target gene. After a false discovery rate adjustment of significant *p* values and a fold change cut off of 1.5 applied, *ALK1* has a significance of *p *= *0.003, ALK3 p* = *0.002, hTGFβ p *= *0.004, LOX p *= *0.002, LTBP3 p *= *0.002, MKP-1 p *= *0.001, and SMAD* also had a significance level *of p*=*0.001.* Non-adjusted *p* values can be found in Table 2. *P* values reflect differences between TG and WT animals within a given gene and heart chamber. Data are expressed as mean ± SEM. Statistical significance represents the genotype/treatment effect comparing TG and WT animals; atrial chamber was included as a repeated factor in the analysis

**Fig. 7:**
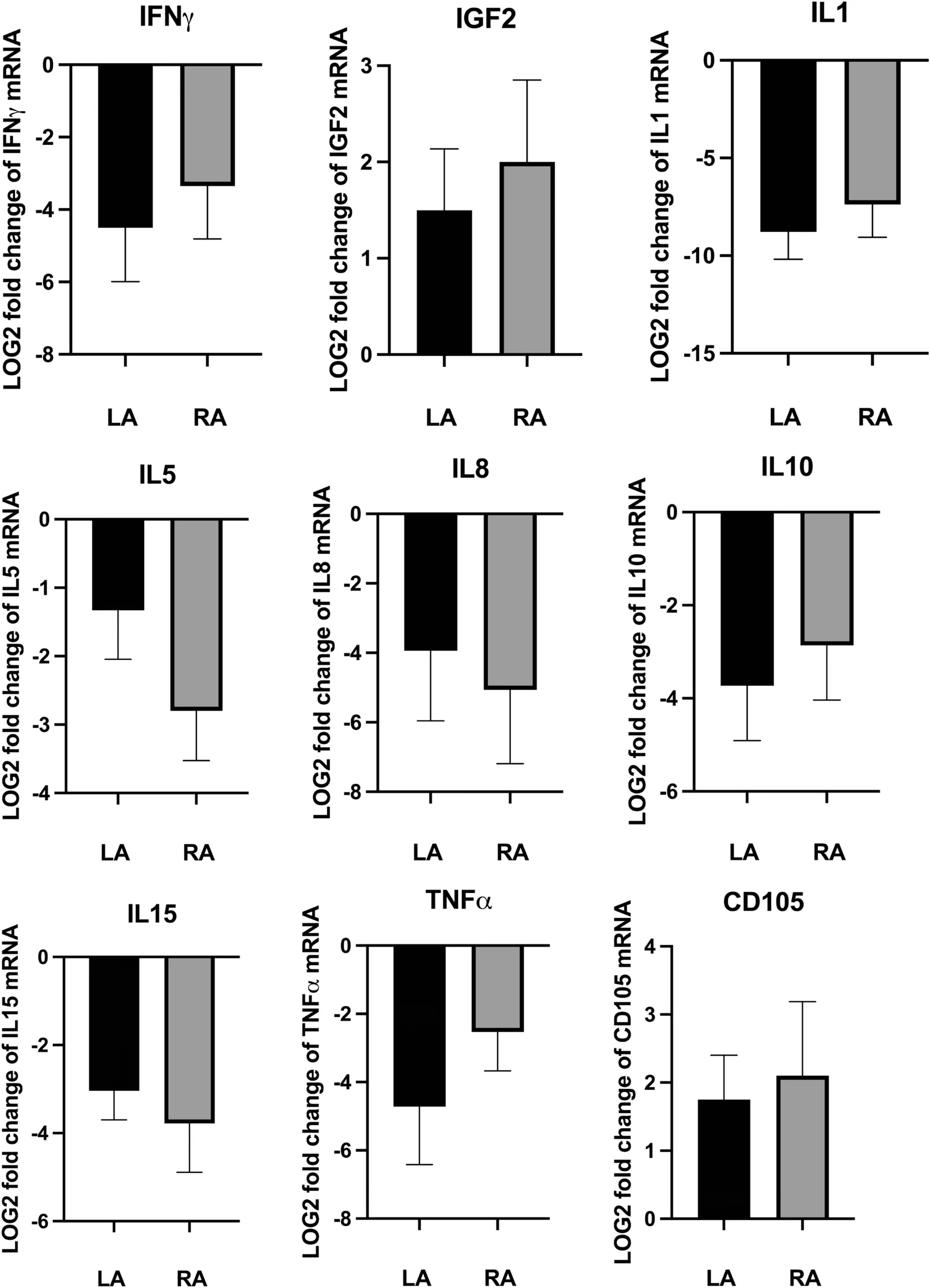
Log2 relative expression of inflammation-related genes in atrial samples showing significant difference between TGF-β1 transgenic (TG; n = 6) and age and sex matched wild-type (WT; n = 6) control goat left (LA) and right (RA) atrial samples. Graphs represent 2^-ΔΔCT fold change in relative mRNA transcript levels in TG animals detected using qRT-PCR, target transcript expression was normalized to the mean mRNA transcript level expression of glyceraldehyde−3-phosphate dehydrogenase (GAPDH) and actin beta (ACTB) and subsequently to the corresponding WT transcript level for the individual target gene. After a false discovery rate adjustment of significant *p* values and a fold change cut off of 1.5 applied, *CD105* had of *p *= *0.002, IFNγ p *= *0.005, IGF2 p *= *0.002, IL1 p *= *0.001, IL5 p *= *0.002,, IL8 p *= *0.001, IL10 p *= *0.004, IL15 P *= *0.003 and TNFα* also had a significance level *of p *= *0.001.* Non-adjusted *p* values can be found in Table 2. *P* values reflect differences between TG and WT aniamls within a given gene and heart chamber. Data are expressed as mean ± SEM. Statistical significance represents the genotype/treatment effect comparing TG and WT animals; atrial chamber was included as a repeated factor in the analysis

**Fig. 8:**
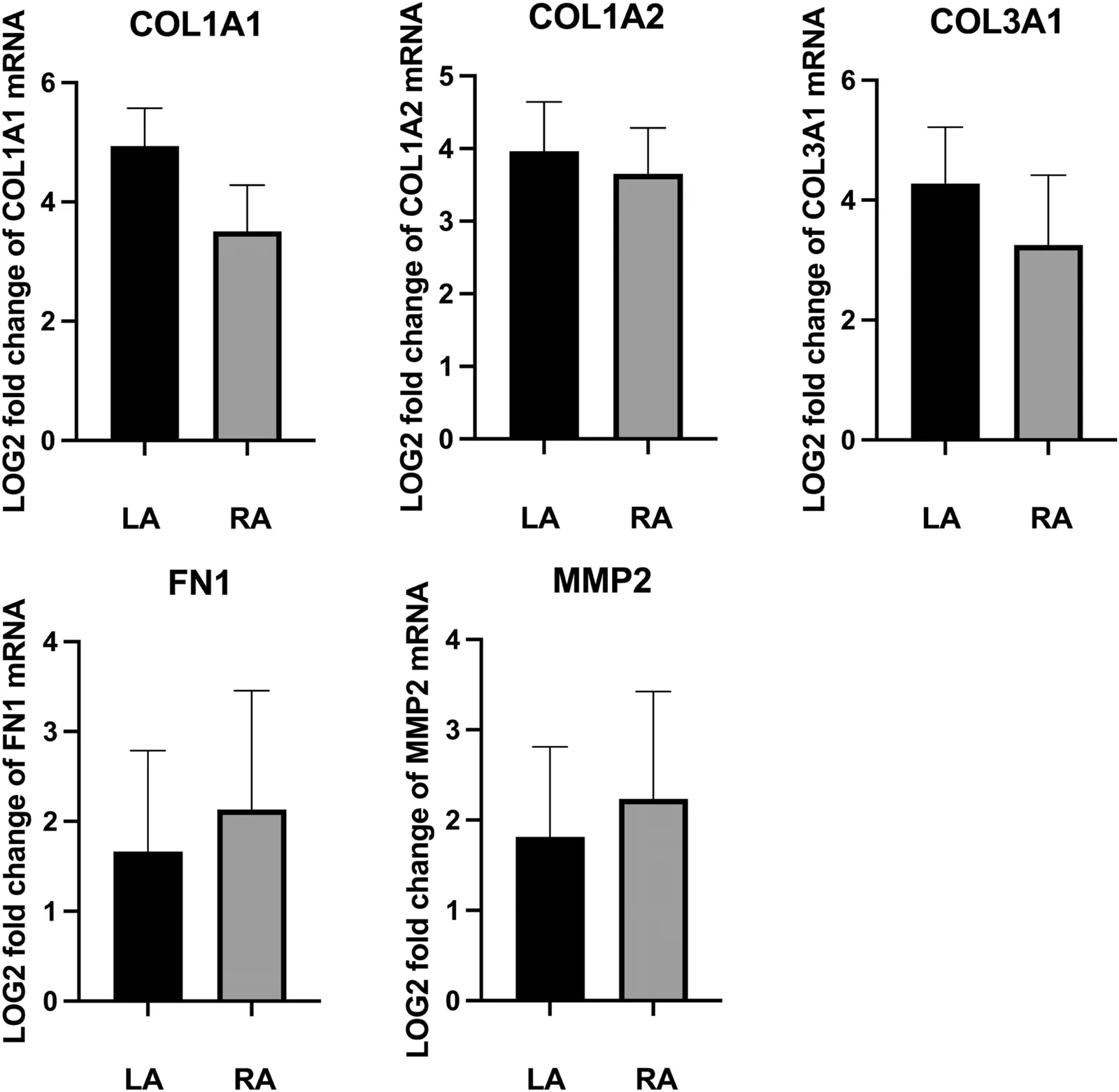
Log2 relative expression of extra cellular matrix related genes in atrial smaples showing significant difference between TGF-β1transgenic (TG; n = 6) and age and sex matched wild-type (WT; n = 6) control goat left (LA) and right (RA) atrial samples. Graphs represent 2^-ΔΔCT fold change in relative mRNA transcript levels in TG animals detected using qRT-PCR, target transcript expression was normalized to the mean mRNA transcript level expression of glyceraldehyde−3-phosphate dehydrogenase (GAPDH) and actin beta (ACTB) and subsequently to the corresponding WT transcript level for the individual target gene. After a false discovery rate adjustment of significant *p* values and a fold change cut off of 1.5 applied, *COL1A1* has a significance of *p *= *0.003, COL1A2 p *= *0.002, COL3A1 p *= *0.002, FN1 p *= *0.005, and MMP2* also had a significance level *of p *= *0.001.* Non-adjusted *p* values can be found in Table 2. *P* values reflect differences between TG and WT animals within a given gene and heart chamber. Data are expressed as mean ± SEM. Statistical significance represents the genotype/treatment effect comparing TG and WT animals; atrial chamber was included as a repeated factor in the analysis

Significant differences between TG and WT goat groups are shown in Figs. 5, 6, 7 and 8. Specifically, *KCNA1, KCNIP1,*and *SHOX2*, in the AF gene category, were all significantly downregulated, while CX43 was upregulated when compared with WT animals. *ALK1, ALK3, hTGF-β, LOX, LTBP3, MKP-1*, and *SMAD* were significantly upregulated in TG animals compared to WT. In the inflammation category, only *CD105* and *IGF2* were significantly upregulated, while *IFNγ, IL1, IL5, IL8, IL10, IL15* and *TNFα* were significantly downregulated in TG animals when compared to WT. Lastly, the ECM category yielded several upregulated genes, including *COL1A1, COL1A2, COL3A1, FN1*, and *MMP2.*

## Discussion

This work builds upon the characterization of a TGF-β1 transgenic goat model of AF. The data show important distinctions between fibrosis and inflammation and their roles in the development and progression of AF. While we have previously established the inducibility and viability of this model (Polejaeva et al. 2016), this work investigates the mechanisms of AF that are not as feasible to investigate with laboratory animal models or human patients. Tissue from goats is similar to humans both in total volume and structure making this model a strong candidate for studying the role fibrosis plays in AF (Kay et al. 1983). This helps elucidate a clearer picture of the pathophysiology of AF, in that, fibrosis without acute myocardial injury is a strong enough trigger to develop and sustain an AF phenotype. As mentioned previously, several genes were differentially expressed between TG and WT animals and seem to have a strong tendency to be associated with an AF phenotype, as discussed in further detail below.

The SMAD family of proteins is part of the TGF-β1 cascade and is implicated in the conversion of an inactive cell type, such as a fibroblast, into an activated myofibroblast cell type (Humeres et al. 2022). *SMAD* and *SMAD3* were upregulated in the TG animals, this is expected as canonical TGF-β1 pathway activation occurs through intracellular SMAD activation (Frangogiannis et al. 2020). Further research is needed to characterize the intricacies the SMAD family may have on TGF-β1 signaling. *ALK1* and *ALK3* are both involved in angiogenesis and cell–cell communication, the upregulation observed in TG goats could be due to new blood vessel formation due to extracellular matrix formation in the atria (Farhan et al. 2022). This gene family is also implicated in TGF-β1 signaling as they are present in cellular membranes and act as binding sites for both TGF-β1 and SMAD proteins for activation of the pathway, as such, increased expression in TG animals is expected (Chen et al. 2016). Evaluating this model of cardiac fibrosis via non-canonical TGF-β1 pathways (i.e. ERK, AKT and MAPK) may be useful to understand if these pathways may contribute to fibrotic signaling in the atria of TG goats.

The bone morphogenic protein (BMP) gene superfamily is closely connected with TGF-β1 and its signaling pathway as they are mostly implicated in cardiac cell differentiation (de Pater et al. 2012). In addition to cellular differentiation, the BMP family is also implicated in embryogenesis, apoptosis, organogenesis, and morphogenesis (Hogan et al. 1996). BMP proteins are part of the SMAD-dependent signaling cascade of TGF-β1 and help bind to ligands that recruit various receptors leading to activation of the LLC of the latent TGF-β1 molecule (Boeuf et al. 2012). We see differential expression with different types of BMP proteins within this family in our study. Specifically, *BMP4* and *BMP6* are involved in dimerization of the activated TGF-β1 molecule (Ye et al. 2023).

As expected, the expression levels of TGF-β1 were increased in TG goats. This aligns with expectations, as TG goats were designed to over-express the molecule. The two variants of latent TGF-β1 binding protein selected as gene targets had a varied response in the TG animals. *LTBP3* is more directly involved in the TGF-β1 pathway and can only bind to the active form of TGF-β1 (Robertson et al. 2015), the increased expression levels we see follow TGF-β1 expression in TG animals.

Collagens are the main constituent of the ECM and are vital to provide scaffolding structure to tissue. However, aberrant, increased levels of collagen can disrupt tissue functions. All three genes related to collagen deposition such as *COL1A1, COL1A2* and *COL3A1* tested in this study had increased expression in the atrium of TG goats compared to WT goats. These genes promote fibrosis through ECM laydown and are linked with AF development (Polejaeva et al. 2016, van den Berg et al. 2021).

*LOX* is another common regulator of ECM. Its upregulation observed here correlates with the involvement in the increased ECM deposition in TG goats as it is found in higher levels in tissues with greater levels of fibrosis (Al–U’datt et al. 2019). Quantifying insoluble ECM fractions in atrial tissue would be a valuable future study to further define fibrotic remodeling in this model of cardiac fibrosis, given Lysyl Oxidase*’*s role (LOX)’ in structural remodeling of the atria. *MKP-1* is a regulator of intracellular signaling pathways and has been implicated in cellular responses to TGF-β1, namely cardiogenesis and the ERK1/2 growth factor pathway, which, in turn, is implicated in cell turnover and fibroblast growth factor activation (Rose et al. 2010). Increased expression levels in TG goats are most likely due to the conversion of inactive fibroblasts into activated myofibroblasts. Fibronectin 1 (*FN1*) also has a strong tie with regulating ECM laydown and has been previously identified to be elevated in human patients with AF (Büttner et al. 2018). Our TG goats exhibiting higher levels of *FN1* than their WT counterparts is expected.

Voltage-gated potassium channels are not traditionally considered drivers of AF; however, an upregulation of potassium channel gene expression has been observed in patients with persistent AF (Glasscock et al. 2015). *KCNIP1* expression is a strong indicator of the development of AF in humans via *KCNIP1*’s association with the L-type calcium current in the heart (Tsai et al. 2016). *KCNIP1* significantly differed between TG and WT animals, with TG goats having a decreased expression level. This is puzzling, as we would have expected increased levels of *KCNIP1.* Further studies, such as CM patch clamping, or fluorescence-activated cell sorting (FACS) to isolate specific cell populations, should be performed to understand any ramifications TGF-β1 may have on ion channels and to understand how changes in cell population may contribute to the observed transcriptional differences. *KCNA1* was found to be significantly different between treatment groups. The downregulation of these genes could show a potential ionic mechanism for AF as a decrease in potassium transport, which impacts the repolarization of the CM, fosters shorter action potential durations causing a lack of arrhythmogenesis (Tsai et al. 2016).

*SHOX2* is a known genetic variant implicated in the development of AF (Hoffmann 2019). Much research is being done in gene and cell therapy to correct AF (Sumer et al. 2020) with *SHOX2* variants. *SHOX2* is vital to the development of the sinoatrial (SA) node and has a large role in the embryogenesis of the cardiac conduction system. Mice with a *SHOX2 -/-* phenotype die at a young age from bradycardia due to a lack of specialized pacemaker cells (Hu et al. 2018). Our gene expression data showed a significant decrease between TG and WT goats, most likely due to a variant of *SHOX2* being expressed. Further research is needed to explain this result. Connexin 43 was significantly upregulated in TG animals when compared to WT goats. Connexins are largely implicated in the electrical signaling of CMs. As non-specific membrane pores, connexins allow small molecules to be transported between cells and have strong ionic conductance allowing for electrical signal transmission between cells (Lambiase et al. 2015). The heart can function because of the electrical signal propagation of these specialized units. Aberrant electrical signals are commonly found in AF, some of which are thought to result from improper connexin signaling either from a decrease in global connexin isoforms or differences in their distribution within the heart (Guo et al. 2022). MMP2 was also upregulated in our TG goat when compared to the WT group and is implicated as a risk factor for recurring AF in humans, particularly when active TGF-β1 is also found to be upregulated and may be implicated in leukocyte transendothelial migration (Guan et al. 2022; Li et al. 2022).

*KCNA1, KCNIP1* and *SHOX2* were significantly downregulated in the TG goats. This is likely due to different variants that would not be picked up by the designed primers. Further research into these expression levels is needed to understand the downregulation in this animal model.

A common cytokine found to be pro-apoptotic is *IFNγ*, this cytokine is commonly found in cancers due to its response to immunological perturbations (Castro et al. 2018). *IFNγ* is largely responsible for leukocyte attraction and cellular differentiation. Mice born without *IFNγ* show immunodeficiencies against bacterial, parasitic, and viral infections (Schroder et al. 2004). Its role in AF is not fully understood, but there is supporting literature for its role in a profibrotic pathway and implicated in cell turnover (Zhou et al. 2021). This supports the finding that TG goats exhibit a significant downregulation of *IFNγ* in cardiac tissue when compared to their WT counterparts, as there were no significant differences found in other genes associated with apoptosis.

Overall, an increase in the gene expression of inflammatory markers, such as interleukins, was not an anticipated result. However, specific interleukin expression may offer some clues into the development of AF in TG goats. AF is thought to have a global inflammatory effect in cardiac muscle, supporting the idea that injury must be present for fibrosis to occur (Oikonomou et al. 2019). Although IL-6 is commonly known as a pro-inflammatory cytokine, it is an anti-inflammatory myokine. That is, when expressed from myocytes, it produces a localized anti-inflammatory effect through stimulating other anti-inflammatory cytokines (Petersen et al. 1985). There is supporting data in human patients showing upregulation in the expression of *IL-6* in patients with lone, paroxysmal, and organic AF (Cheng et al. 2012). Though there is contradictory literature, this highlights the complexity between inflammation and AF and any other factors the overexpression of TGF-β1 could have within the genetically engineered animals.

A previous mouse study shows a lower levels of *IL-1* and *IL-8* in the sera of mice with AF through thromboembolisms (Shan et al. 2019), and the data from our goats agree. *CD105* is a common biomarker predictive of AF when found in atrial tissue, due to its role in angiogenesis post ischemic event in the heart. It is implicated in fibrosis and was found to be upregulated in TG goats compared to their WT counterparts (Gramley et al. 2010). Insulin-like Growth Factor 2 (*IGF2*) is found in a myriad of fibrotic diseases (e.g. idiopathic pulmonary fibrosis, scar formation, liver, and renal fibrosis). The upregulation we found in TG goats is expected as one of its roles in a fibrotic disease state is excessive ECM deposition (Zhu et al. 2022). Our results align with expectations with these upregulated genes and show that inflammation itself is not the primary driving factor for the development of AF in TG animals.

The lack of significance in apoptotic and decreased expression of inflammatory genes was unsurprising as no goats had any cardiac insult other than pacemaker implantation. Although cell death and inflammation are implicated in AF and can facilitate fibrosis (Hu et al. 2015), our results are as expected as the fibrosis seen in TG animals is likely due to excess and active TGF-β1 expression. Acute inflammation, such as post operative AF (Heijman et al. 2020) would be an unlikely candidate for fibrosis found in these animals as no experimental parameters would have introduced a sudden change to the substrate of the heart, such as an ablative intervention or other procedure. Though pacemaker implantation may have induced a localized increase of inflammatory markers, no data were collected from the animals at that time. Chronic inflammation can occur when a continuous insult happens to cardiac tissue. Although certainly a common driver for AF development, typically chronic inflammation is found as a comorbidity with AF in ischemic heart failure (Li et al. 2021).

AF is a disease with many origin points. Not all known genetic associations should be expected to be present in a transgenic goat model of cardiac fibrosis. Though perplexing that not all TGF-β1 pathway-related genes investigated were upregulated, there are many nuances in the regulation of the pathway, namely regulation of fibroblast activation through the SMAD protein family, that could account for the lack of differential expression between treatments (Gu et al. 2013). Another potential factor contributing to the lack of significance in some genes could be because both goat groups ranged in age from three to six years; as fully mature and older animals, fibrosis could be increased simply due to age (Biernacka et al. 2011). We investigated this with an unpublished study assessing fibrosis levels in aged WT and TG goats, finding no correlation with fibrosis in aging goat hearts in either unmodified or modified hearts.

This study shows that a large animal model of cardiac fibrosis can help elucidate the effects of fibrosis on AF. These data support our previous findings that fibrosis expressed in the atria of TG goats is likely to come from TGF-β1 overexpression in its active form rather than an inflammation background, and that fibrosis can sustain AF inducibility. The data shown here is difficult to extrapolate to humans as the typical clinical presentation of AF does not come with overexpression of TGF-β1. The TG goats were generated using an α-MHC promoter to drive cardiac-specific expression of active human TGF-β1. Because the present study was designed to investigate the atrial substrate underlying AF, the molecular, histologic, and inflammatory analyses presented here focus on atrial tissue. The present study did not directly assess TGF-β1 expression or transcriptional remodeling in ventricular tissue. Performing more in depth electrophysiological studies, such as optical mapping, or patch clamping cardiomyocytes, should be explored to gain more understanding of the cellular behavior within a fibrotic substrate and how cellular communication is impacted. Targeting more clinically relevant genes to generate different large animal models could further contribute to improve the treatment of this condition in clinical practice. Further investigation at the molecular level, such as utilizing single cell RNA sequencing, *in* situ hybridization, or isolating and sorting cardiomyocytes may allow for even more insight into the role of fibrosis in AF, such studies should be performed to fully elucidate this effect. If additional clinically relevant large animal models are developed, a better understanding of the genesis and perpetuation of AF can be understood. Broader transcriptomic approaches such as RNA sequencing could identify additional pathways not captured by this targeted approach and would provide a more comprehensive assessment of the molecular response to TGF-β1 overexpression. Furthermore, because the transcriptional analyses were performed in animals with established fibrosis and AF susceptibility, the present study cannot determine whether the observed transcriptional changes are direct consequences of TGF-β1 overexpression, secondary to structural remodeling, or associated with the AF phenotype itself.

As all animals in the study were female, findings should be interpreted in the context of female cardiac biology and not be directly generalized to male animals. Although experiments were conducted during anestrous and all animals were more than one year post-parity, reproductive hormonal status among animals may have contributed to inter-animal variability in gene expression. Moreover, the TG and WT animals have some differing breeds in their genetic background. Although age and sex matched WT animals were used as controls, we cannot completely exclude an influence of genetic background on the observed phenotypes or transcriptional profiles. Future studies using both sexes and genetically matched control would strengthen attribution of these differences as they relate to TGF-β1 overexpression.

More work should be done to characterize the full effect that TGF-β1 induced fibrosis has on cardiac tissue and how that shapes the conductivity of the heart in clinical practice. More refined gene expression could be performed based on the data presented here in order to fully elucidate the mechanisms behind the TGF-β1 cascade in these goats and what effect the transgene has on the phenotype of these animals. This study exhibits how cardiac substrate influences conductivity, and therefore, functionality of the heart. Tissue models have shown that uneven deposition of fibrosis can lead to functional barriers of electrical signal, prompting an increase in self-sustaining re-entrant electrical patterns, promoting arrythmogenesis (Colman et al. 2023). Further, external physiologic factors may serve as a “second hit”, or trigger for sustained AF episodes with a fibrotic substrate present as our group showed with endurance exercise in this goat model (Regouski et al. 2019). A fibrotic, or poorly conducting atria may not be enough to produce an arrythmia spontaneously, but coupled with factors such as exercise or aging may present enough overload to promote an arrhythmogenic state. Increased structural anisotropy may shed more light into the substrate plus trigger concept routinely discussed in atrial fibrillation research. The heterogeneity of atrial fibers, differences in fiber direction leading to disorganized electrical conduction is found in the TG goats used in this study which may explain why some animals are more inducible than others, particularly with sustained AF episodes (Kwan et al. 2024). This insight may exhibit how disorganized atrial tissue leads to a predisposition to AF, while other “triggering” events may promote the arrhythmia, such as exercise. Looking at ion channels via cardiomyocyte patch clamping may be helpful to understand if an over-expression of TGF-β1 alters these specialized cells themselves, or if the AF observed in these animals comes strictly from excessive fibrosis in the atria. Further studies investigating specific effects from environmental triggers may help contribute to the overall understanding of the development of AF and could be extrapolated to humans.

This body of work showcases the development of a transgenic large animal model of cardiac fibrosis, the first of its kind. There is also strong evidence that structural remodeling on its own without insult or injury can initiate and sustain an AF phenotype without constant perturbation by inflammation, proving important to understand some components of the development of the disease.

## Data Availability

All data supporting the findings of this study are available within the paper and its Supplementary Information.
